# Statins and long-term risk of revision surgery after total hip arthroplasty in osteoarthritis: a multi-source data linkage study

**DOI:** 10.3389/fphar.2025.1492200

**Published:** 2025-04-15

**Authors:** Andrea D'Amuri, Barbara Bordini, Mauro Pagani, Jacopo Ciaffi, Claudio D'Agostino, Alberto Di Martino, Cesare Faldini, Francesco Ursini

**Affiliations:** ^1^ Unit of Internal Medicine, Azienda Ospedaliera Carlo Poma, Mantova, Italy; ^2^ Laboratory of Medical Technology, IRCCS Istituto Ortopedico Rizzoli, Bologna, Emilia-Romagna, Italy; ^3^ Medicine and Rheumatology Unit, IRCCS Istituto Ortopedico Rizzoli, Bologna, Emilia-Romagna, Italy; ^4^ Department of Biomedical and Neuromotor Sciences, University of Bologna, Bologna,Emilia-Romagna, Italy; ^5^ 1st Orthopedic and Traumatology Department, IRCCS Istituto Ortopedico Rizzoli, Bologna, Emilia-Romagna, Italy

**Keywords:** osteoarthritis, statins, orthopedic surgery, total hip arthroplasty, prosthesis outcomes

## Abstract

**Background:**

Statins, widely used lipid lowering drugs, have been associated with pleiotropic beneficial effects. Notably, studies conducted *in vitro* and *in vivo* suggest a link between statins and bone metabolism. Observational data in humans also hint at a decreased fracture rate among statin users. Revision of total hip arthroplasty (THA) is a serious and costly medical event. Whether statins might influence THA failure is not clear. Aim of the current study is to assess how the preoperative use of statins may influence the risk of THA revision in patients with hip osteoarthritis (OA).

**Methods:**

We performed a retrospective analysis of patients who underwent THA for OA in the Italian RIPO registry of Emilia-Romagna. Electronic health records were scrutinized to gather information regarding comorbidities and statin prescriptions. We employed propensity score (PS) matching to pair 1:1 statin users (SU) with statin non-users (SNU), considering factors such as age, sex, and the duration of follow-up. Survival of THA was compared between the two groups; secondary analyses were performed to ascertain the role of mortality, sex, indication for statin treatment, and statin potency or lipophilicity.

**Results:**

10,927 patients were classified as SU and PS-matched with SNU. SU showed a reduced risk of THA revision over a 15-year period (adjHR 0.76, 95% CI: 0.67–0.88; p < 0.001). Notably, this observation remained consistent regardless of the indication for statin therapy or the specific characteristics of the statin medications prescribed, and it was more pronounced among male patients (adjHR 0.64, 95% CI: 0.52–0.80, p < 0.001).

**Conclusion:**

Our findings suggest that statin treatment is associated with a decreased risk of long-term THA revision in patients with OA, irrespective of the original indication for statin therapy.

## 1 Introduction

Statins are lipid-lowering agents that work by inhibiting the enzyme 3-hydroxy-3-methylglutaryl coenzyme A (HMG-CoA) reductase. These cost-effective drugs are widely prescribed to lower cholesterol levels in patients with dyslipidemia who are at risk for cardiovascular disease (Walley et al., 2004). In addition to their well-known impact on hepatic lipoprotein production, growing evidence suggests that statins possess anti-inflammatory and immunomodulating properties, leading to a multitude of pleiotropic actions. These include enhancing atherosclerotic plaque stability, modulating the host response to infections, and potentially affecting the development of diseases such as cancer, Parkinson’s disease, and dementia (Mohammad et al., 2019). Furthermore, *in vitro* studies have shown potential bone-protecting effects of statins by influencing the proliferation, differentiation, and protection of osteoblasts, while also reducing osteoclastogenesis (Oryan et al., 2015).

Total hip arthroplasty (THA) is one of the most commonly performed elective orthopedic surgical procedures, with a continuously growing trend (Kurtz et al., 2007). On the other hand, the number of THA is inevitably accompanied by a rising demand for implant revisions, which represent a serious and undesirable outcome, carrying significant risks to patients and substantial costs to the healthcare systems (Ashkenazi et al., 2023). The need for revision of THA can arise from different potential causes of failure, including aseptic loosening of components, infections, dislocation, and periprosthetic fractures (Havelin et al., 2000). Among these, aseptic loosening is a major issue, being responsible for 35.8% of overall primary THA implant failure requiring revision surgery (RIPO database, 2025) and results from both physical factors and an imbalance in the cellular response to the implant, which favors osteolysis over bone formation.

In this regard, preclinical studies suggest that simvastatin enhances the expression of bone morphogenetic protein-2 (BMP-2), a potent stimulator of osteoblast differentiation and activity, promotes mineralization in cultured osteoblasts, and inhibits osteoclastic differentiation, thus producing a net anabolic effect on bone (Maeda et al., 2001; Granat et al., 2024; Yamashita et al., 2010). Further, in osteoporotic mice, statins facilitate osseointegration of titanium implants by improving bone-implant contact, bone mineral density (BMD), and bone volume around implants (Pruthi et al., 2023). Additionally, in a murine calvarial model of aseptic loosening, simvastatin promoted bone formation, indicating a potential effect in mitigating wear debris-induced osteolysis after arthroplasty (Von Knoch et al., 2005a). In support of the beneficial effect of statins on bone metabolism, a meta-analysis of clinical studies published in 2017 concluded that statin treatment may be associated with a reduced risk of overall and hip fractures, as well as increased BMD at the total hip and lumbar spine (An et al., 2017). On the other hand, *post hoc* analyses of clinical trials with primarily cardiovascular endpoints failed to show any significant difference in fracture rates between patients taking statins and those taking placebo (Reid et al., 2001; Orbach, 2000). It’s worth noting that the clinical fracture rates in these cardiovascular trials were very low. Therefore, the currently available clinical evidence, although suggesting a potential positive effect on bone, remains incomplete and inconsistent.

However, some previous studies have suggested that statin treatment may have a favorable impact on both medical and surgical outcomes following THA (Baghdadi et al., 2024). To provide an additional contribution to this topic, we conducted a multi-source data linkage study aimed at evaluating the effects of preoperative statin use on the risk of THA revision in patients with hip osteoarthritis (OA).

## 2 Materials and methods

### 2.1 Aim of the study

The primary aim of the study was to evaluate if statin treatment is associated with the risk of THA revision in patients with hip OA. Secondary analyses were performed to account for sex differences, competing risk of death, indication for statin treatment, statin potency or lipophilicity.

### 2.2 Population

We performed a retrospective data linkage analysis, starting from data collected in the Emilia-Romagna Orthopedic Arthroplasty Implants Register (RIPO) (IRCCS Istituto Ortopedico Rizzoli, 2025). RIPO systematically collects information from hip, knee, and shoulder arthroplasty procedures carried out in 62 orthopedic departments, both public and private, located in the Emilia-Romagna region (northern Italy) and received formal approval from the regional government assembly (Emilia-Romagna Regional Law 1 June 2017, No. 9, Chapter III, Article 6). The population of this area is about 4.5 million and the registry reaches a capture rate of around 95% (IRCCS Istituto Ortopedico Rizzoli, 2025). The design of RIPO is aligned with international standards and the registry is affiliated with the International Society of Arthroplasty Registries allowing for meaningful comparisons with other major national registries worldwide (ISAR - International Society of Arthroplasty Registries, 2025). It’s worth noting that the RIPO registry also covers data for Emilia-Romagna residents who receive revision surgery outside the region. This is because, within the Italian National Health System (Sistema Sanitario Nazionale, SSN), all surgical procedures performed anywhere in Italy are documented and billed back to the patient’s region of residence.

The information included in the RIPO registry for each patient undergoing arthroplasty, whether primary or revision surgery, comprises details such as age, sex, body mass index (BMI), the patient’s clinical history, the clinical reason for surgery, specifics of the implant model and design, the surgeon responsible for the procedure, and the hospital where the surgery took place. Data are entered by the operating surgeon at the conclusion of each procedure. However, it’s important to mention that postoperative care, the rehabilitation process, and clinical scores are not part of the collected data. To ensure a uniform and comparable sample, we excluded from the analysis subjects who had undergone THA for hip OA with cemented implants and those receiving large (≥36 mm) head with metal-on-metal bearing surfaces. This *a priori* exclusion of cemented implants is due to the fact that cemented implant are not supposed to integrate with bone, and these are designed to improve implant stability in a context of known poor bone metabolism (Blankstein et al., 2020); on the other side, metal-on-metal coupling THA implants were excluded because they carry an intrinsic higher risk of implant failure owing to poor tribology, and their outcomes are routinely removed from current registry studies dealing with THA implants survival (Di Martino et al., 2021; Tierney et al., 2023).

**FIGURE 1:**
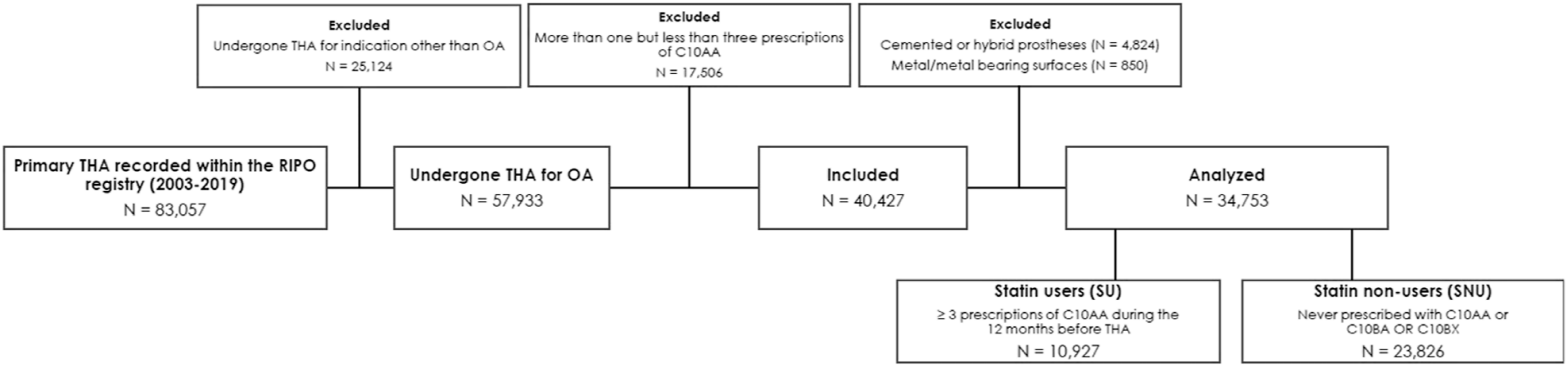
Selection process and causes of exclusion according to prespecified inclusion and exclusion criteria. Legend: OA, osteoarthritis; RIPO, Emilia-Romagna Orthopedic Arthroplasty Implants Register; THA, total hip arthroplasty.

The data analysis covered the period from January 2003 to December 2019. The selection process is outlined in Figure 1. All sensitive data were carefully processed in a pseudo-anonymized format, with all personally identifiable information removed.

The integration between the RIPO registry and other administrative databases was ensured by the automatic assignment of a unique and anonymous identification number (*PROG_PAZ*) by the Information System for Health Policies and Social Policies (SISEPS, Sistema Informativo Politiche per la Salute e Politiche Sociali) to all residents who have had at least one contact with the Emilia-Romagna health service. This identifier is systematically reported in any data source and enables record linkage across databases while protecting patient anonymity.

Ethical approval for the study was not required as registry studies are covered by the informed consent signed at treatment. The study complies with the Declaration of Helsinki and its latest amendments.

### 2.3 Identification of statin users

In Italy, individuals with chronic illnesses are entitled to receive free drug dispensation through the SSN. To identify statin users, we conducted a cross-reference with the regional Pharmaceutical Territorial Assistance (PTA) database. This database systematically records information for each prescription of drugs directly provided by the SSN. The PTA database employs the Anatomical Therapeutic Chemical (ATC) classification system, which assigns a code to each drug, identifying both its pharmacological class and its specific active ingredient.

The HMGCoA-reductase inhibitors are classified under the code C10AA, with a progressive number from 01 to 08 to specify the specific molecule: C10AA01 for simvastatin, C10AA02 for lovastatin, C10AA03 for pravastatin, C10AA04 for fluvastatin, C10AA05 for atorvastatin, C10AA06 for cerivastatin, C10AA07 for rosuvastatin, C10AA08 for pitavastatin. Additionally, the identifier C10BA includes combinations of a statin with other lipid-lowering agents such as ezetimibe, while C10BX covers combinations of a statin with antihypertensive or antiplatelet drugs.

We defined as statin users (SU) those individuals who received at least three prescriptions with C10AA codes in the year preceding their THA procedure. We chose three prescriptions because this represents a treatment duration of at least 3 months, an exposure that has been associated with THA outcomes in a previous study (Thillemann et al., 2010). The statin non-users (SNU) group comprised individuals who did not have any C10AA, C10BA, or C10BX prescriptions in the year prior to their THA and throughout the entire follow-up period. Together with the categorical variable identifying the ATC code of the drug (primary identifier), the PTA database includes a string variable containing additional information about the specific prescription, such as the brand name, the number of tablets/capsules per package, and the dosage.

Therefore, we conducted additional analyses based on statins potency and lipophilicity. Given that the lipid-lowering effects of statins depend on the type of statin and its dosage, statins are commonly classified as high-potency statins (e.g., atorvastatin 40 mg or rosuvastatin 20 mg), low-potency statins (e.g., simvastatin 10 mg, pravastatin 20 mg, lovastatin 20 mg, fluvastatin 20 mg, pitavastatin 1 mg), and moderate-potency statins (the same molecules at remaining dosages) (Stone et al., 2014). Regarding lipophilicity, a chemical property that could impact clinical effects by affecting the passage through cellular membranes, statins were divided into hydrophilic (rosuvastatin and pravastatin) and lipophilic (all the remaining molecules) (Climent et al., 2021). For the assignment of the patient to the potency/lipophilicity group, in case of use of different statins during the prespecified period, the last prescribed molecule before surgery was used.

### 2.4 Identification of patients receiving statins for primary vs. secondary cardiovascular prevention

Statins are a class of drugs primarily used to lower lipid levels and manage dyslipidemia, ultimately reducing the risk of cardiovascular events. However, the specific indication for statin therapy can vary, depending on whether it is intended for primary cardiovascular prevention (CVP) or secondary CVP. Primary CVP involves treating individuals with high cholesterol levels who have not yet experienced cardiovascular, cerebrovascular, or peripheral artery diseases. Secondary CVP, on the other hand, targets patients who have already suffered from ischemic events, requiring different statin potency and more aggressive lipid targets (Mach et al., 2020). Diabetes presents a unique clinical scenario characterized by a significantly elevated cardiovascular risk. In many cases, this risk is very close to that of individuals in the secondary CVP category. Therefore, patients with diabetes often require statin treatment with lipid targets that are as stringent as those for secondary CVP (Marx et al., 2023).

To assess whether the outcomes were influenced by patients’ comorbidities, we conducted a cross-referencing analysis using national administrative hospital discharge records. Specifically, we classified subjects with a clinical history of coronary heart disease (CHD), cerebrovascular disease (CeVD), peripheral artery disease (PAD), or diabetes as being in the secondary CVP category. CHD and CeVD were identified using International Classification of Diseases (ICD-9) codes recommended by the Italian Health Authority (Istituto Superiore di Sanità - ISS). Codes associated with fatal events or hemorrhagic strokes were excluded (Il Progetto Cuore, 2025). Consequently, individuals were categorized as having CHD if they had a history of at least one hospital discharge with ICD-9 codes related to ischemic heart disease (ICD-9 codes 410-414) or coronary revascularization procedures, whether endovascular (ICD-9 codes 36.01, 36.02, 36.05, or 36.06) or surgical (ICD-9 codes 36.1). A history of CeVD was attributed to subjects with at least one hospital discharge containing ICD-9 codes related to ischemic stroke (ICD-9 codes 434-436). Subjects were considered to have PAD if they had at least one hospital discharge with ICD-9 codes related to lower limb atherosclerotic disease (ICD-9 codes 440.20-440.24 or 440.29). This method, which has been previously reported in the literature, has proven to be straightforward yet effective, with a sensitivity of 76.9% and specificity of 89.3% (Fan et al., 2013).

As for diabetes status, we defined diabetic subjects as those with either a hospital discharge containing ICD codes related to diabetes (ICD-9 codes 250) or a prescription for anti-diabetic drugs listed in the PTA registry (ATC codes A10), in line with established practices in the literature (Lipscombe et al., 2018).

Subjects who did not meet the criteria for secondary CVP were categorized as being in the primary CVP group.

### 2.5 Statistical analysis

Data are presented as mean (range) or number (percentage) as appropriate. For comparison, we generated a 1:1 matched cohort with the SU group from the individuals who underwent primary OA enrolled in the RIPO registry, using the propensity score (PS), as previously described (Austin, 2010). The covariates entered in the score were sex, age class at THA and follow-up duration.

Continuous variables were compared between groups using Student’s t-test; Fisher’s exact test was used to detect differences in dichotomic variables. The survival rates of implants were calculated and plotted according to the Kaplan-Meier method. The main outcome was surgical revision, defined as the removal or change of any component of the implant. Implants were followed until the last date of observation (date of death or 31st December 2021). The log-rank test was employed to detect differences between survival curves. A multivariate Cox regression model was used to estimate age- and sex-adjusted hazard ratio (adjHR) and corresponding 95% confidence interval (95% CI). The proportional hazards assumption was tested by the Schoenfeld residual method. The proportional hazards assumption was evaluated using the Schoenfeld residual method.

The adjHR is a commonly used measure in survival analysis to compare the risk of the event of interest (revision surgery) between two groups (SU vs. SNU) at any given time, while accounting for potential confounders (age and sex) known to influence the outcome. Age and sex were chosen as covariates based on previous studies using RIPO data, which identified them as the main determinants of revision risk (Boyer et al., 2019). Other variables, such as BMI, were not included, as they have not shown a significant association with the risk of revision in this dataset (Affatato et al., 2019). The Wald test was used to calculate the p-values for data obtained from the Cox multiple regression analysis.

Further, as death changes the probability of a patient’s prosthesis being revised, we performed a cumulative incidence competing risk analysis, treating death-from-any-cause as the competing event of interest. Revision rates were then compared between two or more groups using the Gray’s test.

Statistical analyses were performed with the use of JMP^®^, Version 12.0.1 (SAS Institute Inc., Cary, NC, 1989–2007). and R version 3.4.2. (Comprehensive R Archive Network), with statistical significance defined as p < 0.05 (R: The R Project for Statistical Computing n.d. https://www.r-project.org/; accessed 2 January 2023).

## 3 Results

### 3.1 Characteristics of the study cohort

**FIGURE 2:**
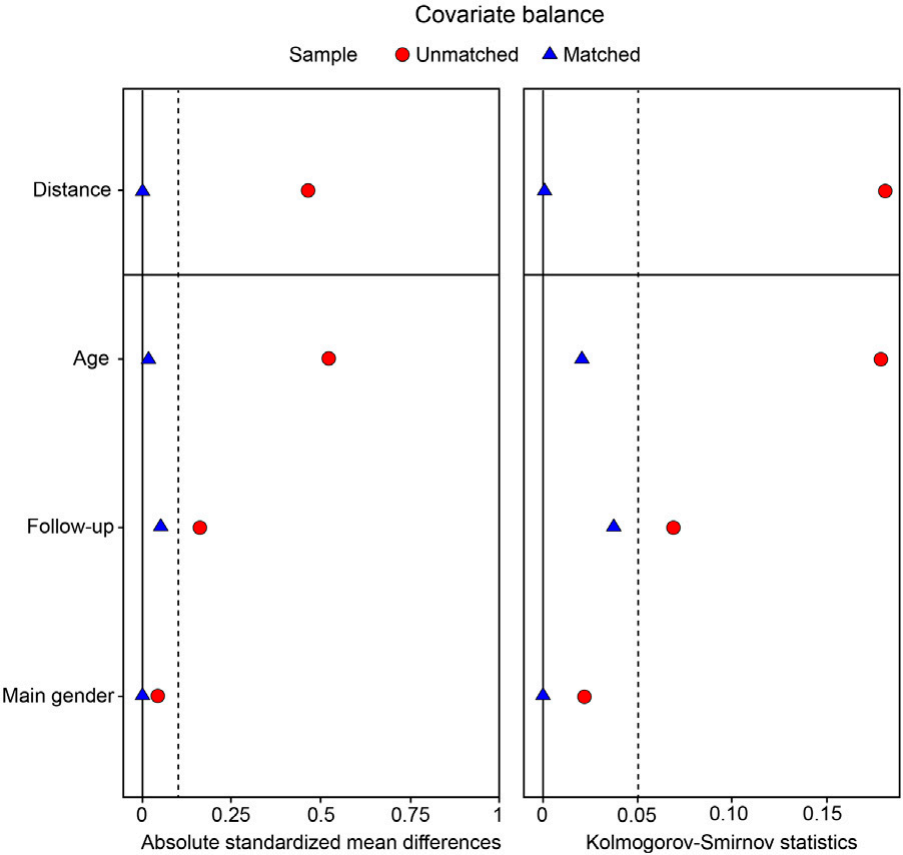
Love plot summarizing covariate balance statistics (absolute standardized mean differences, Kolmogorov-Smirnov test) before and after propensity score (PS) matching.

We initially screened a total of 83,057 patients enrolled in the RIPO registry who had undergone THA. Following the prespecified criteria, we excluded 25,124 individuals who had undergone THA for reasons other than OA. Subsequently, we eliminated 5,674 patients who received cemented or metal-on-metal prostheses and 17,506 patients who did n’t meet the criteria for either SU or SNU definitions, as depicted in Figure 1. Following this selection process, we identified 10,927 individuals as SU and 23,826 as SNU. For the purpose of the analysis, we used PS-matching for pairing 10,927 SU with an equal number of SNU. Balance diagnostics for the variables used are illustrated in Figure 2.

Before matching, SU were more commonly females (56.6% vs. 54.4%, p < 0.001) and older than SNU, with an average age of 73 years (rage 36–96) compared to 69 years (range 18–96, p < 0.001). After matching, the average age was 73 years (range 36–96) for SU and 73 years (range 41–94) for SNU, with females accounting for 56.6% of cases in both cohorts. The number of individuals classified as overweight or obese based on BMI was higher in the SU group (p < 0.001) both before and after matching. The mean follow-up duration was slightly, although significantly, shorter for SU compared to SNU [7.1 years (range 0.0–18.9) vs. 7.3 years (range 0.0–19.0), p < 0.001]. Table 1 provides an overview of the baseline characteristics of the study cohorts.

**TABLE 1:** General characteristics of the study cohorts.

	Statin users (SU) N = 10,927	PS-matched statin non-users (SNU) N = 10,927	P value
Age, mean (range)	73 (36–96)	73 (41–94)	0.2
Female sex, n (%)	6,185 (56.6)	6,185 (56.6)	>0.9
Mean follow-up years (range)	7.1 (0.0–18.9)	7.3 (0.0–19.0)	<0.001
BMI category, n (%)			<0.001
Underweight, n (%)	28 (0.3)	71 (0.8)	—
Normal weight, n (%)	2,283 (24.7)	2,773 (29.7)	—
Overweight, n (%)	4,437 (48.0)	4,373 (46.8)	—
Obese, n (%)	2,501 (27.0)	2,121 (22.7)	—
Indication for statin prescription			
Primary prevention, n (%)	4,661 (42.7)	—	—
Secondary prevention, n (%)	6,266 (57.3)	—	—
LOW-potency statin, n (%)	571 (5.2)	—	—
MODERATE-POTENCY STATIN, N (%)	9,461 (86.6)		
HIGH-POTENCY STATIN, N (%)	895 (8.2)		
Lipophilic statin, n (%)	8,447 (77.3)	—	—
Comorbidities			
T2D, n (%)	5,676 (51.9)	2,089 (19.1)	<0.001
CHD, n (%)	2,213 (20.3)	274 (2.5)	<0.001
CeVD, n (%)	223 (2.0)	175 (1.6)	0.02
PAD, n (%)	177 (1.6)	39 (0.4)	<0.001
Number of CVD risk factors			
One, n (%)	4,124 (37.7)	2,098 (19.2)	<0.001
Two, n (%)	1,983 (18.1)	214 (1.9)	<0.001
Three or more, n (%)	159 (1.4)	18 (0.2)	<0.001

**Legend:** BMI, body mass index; CeVD, cerebrovascular disease; CHD, coronary heart disease; CVD, cardiovascular disease; PAD, peripheral artery disease; PS, propensity score; T2D, type 2 diabetes.

The vast majority of patients (10,632, 97.3%) in the SU group continued statin therapy 1 year after THA; 8,987 (82.2%) had a statin prescription in the 12 months preceding the event (revision) or censoring. Of these, 1,415 (15.7%) patients switched to a statin with a different potency during follow-up, with 1,074 (75.9%) transitioning to a more potent statin (low to moderate potency or moderate to high potency).

### 3.2 THA survival

During the follow-up period, we documented a total of 819 cases of THA revisions, of which 351 occurred in SU and 468 in SNU. Individual causes of THA revision are detailed in Table 2.

**TABLE 2:** Causes of arthroplasty failure.

Causes of failure	Statin users group					
	NO			Yes		
	No	% tot	% fail	No	% tot	% fail
PERIPROSTHETIC FRACTURE	109	1.0	22.9	64	0.6	17.8
ASEPTIC STEM MOBILIZATION	87	0.8	18.3	69	0.6	19.2
DISLOCATION	57	0.5	12.0	48	0.4	13.3
ASEPTIC CUP MOBILIZATION	43	0.4	9.1	34	0.3	9.4
PROSTHESIS BREAKAGE	48	0.4	10.1	29	0.3	8.1
SEPTIC MOBILIZATION	29	0.3	6.1	23	0.2	6.4
PRIMARY INSTABILITY	11	0.1	2.3	13	0.1	3.6
PAIN (without component mobilization)	10	0.1	2.1	12	0.1	3.3
GLOBAL ASEPTIC MOBILIZATION	11	0.1	2.3	7	0.1	1.9
POLY WEAR	7	0.1	1.5	3	0.0	0.8
HETEROTOPIC OSSIFICATIONS	3	0.0	0.6	6	0.1	1.7
METALLOSIS	1	0.0	0.2	1	0.0	0.3
OTHERS	9	0.1	1.9	21	0.2	5.8
LOSS DATA	50	0.5	10.5	30	0.3	8.3
TOTAL	475	4.3	100.0	360	3.3	100.0

**FIGURE 3:**
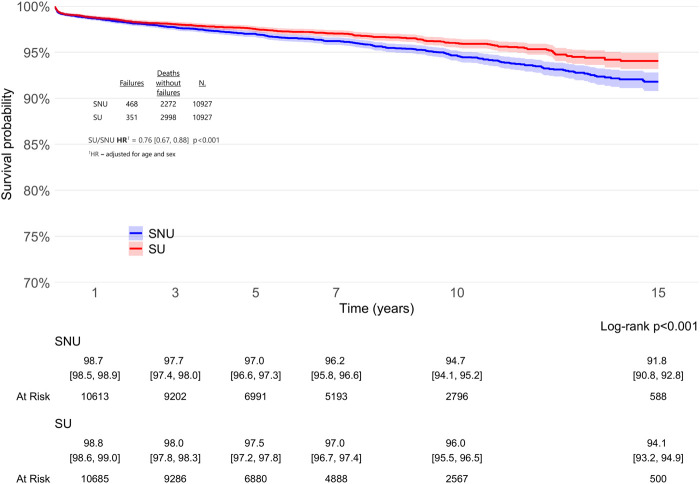
Survival rates of total hip arthroplasty (THA) in statin users (SU) vs. statin non-users (SNU) plotted according to the Kaplan-Meier method (main outcome: surgical revision).

**FIGURE 4:**
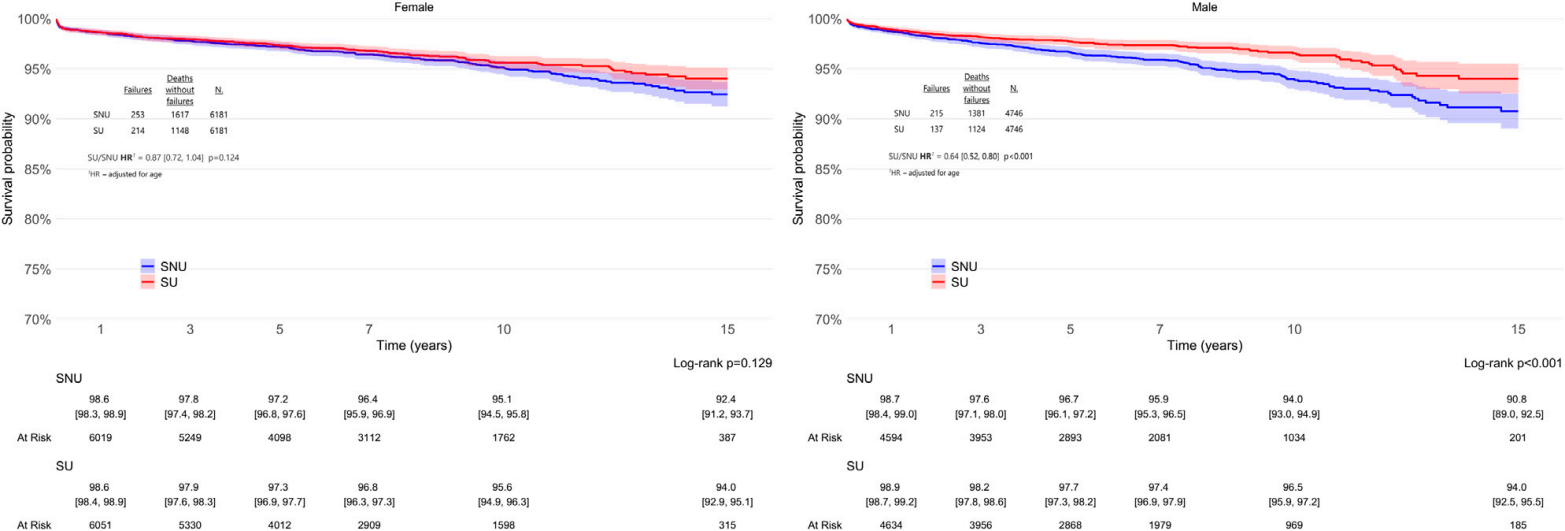
Survival rates of total hip arthroplasty (THA) in statin users (SU) vs. statin non-users (SNU) plotted according to the Kaplan-Meier method (main outcome: surgical revision) and stratified according to sex.

Analysis of the cohorts revealed that SU exhibited a significantly lower risk of THA revision when compared to PS-matched SNU over a 15-year period (adjHR 0.76, 95% CI: 0.67–0.88; p < 0.001), as detailed in Figure 3. The global Schoenfeld residual test was used to check the proportional hazard (PH) assumptions, and it was violated (p value <0.05). Therefore, a stratified Cox model was considered more appropriate for this data; further, sex can influence arthroplasty outcomes, with males having a 33% higher risk of revision after THA compared to females (Towle and Monnot, 2016). When patients were stratified according to sex (Figure 4), the difference in THA survival remained statistically significant in the male population only (adjHR 0.64, 95% CI: 0.52–0.80, p < 0.001) although a trend was evident also in the female population (adjHR 0.87, 95% CI: 0.72–1.04, p = 0.12). The global Schoenfeld test p-values were 0.13 and 0.29 for the female and male stratified Cox models, respectively, further supporting a good fit between the Cox model and the stratified dataset.

**FIGURE 5:**
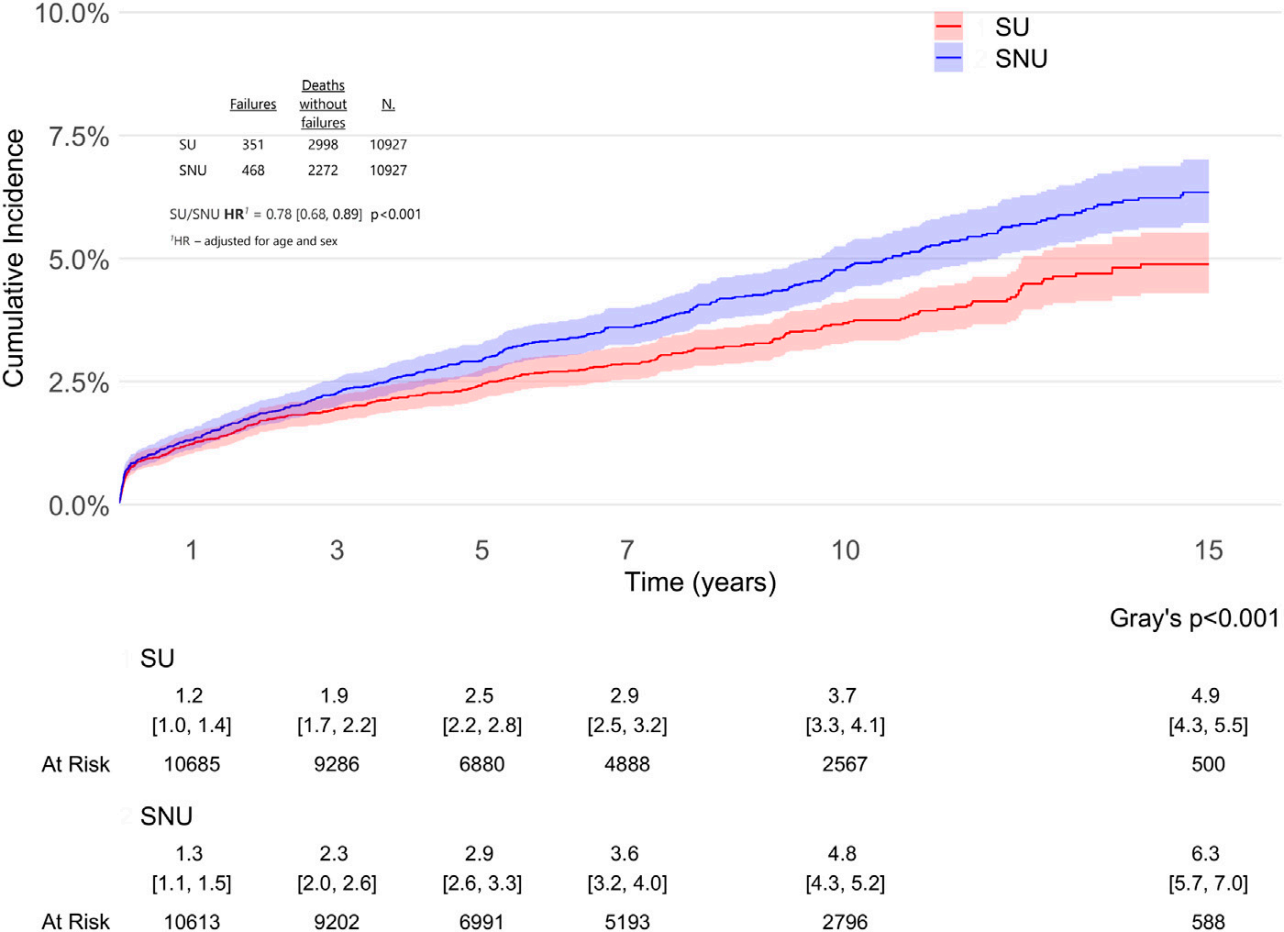
Cause-specific cumulative incidence of total hip arthroplasty (THA) revision in statin users (SU) vs. statin non-users (SNU) accounting for competing risk of death.

The difference in risk of THA revision in SU vs. PS-matched SNU remained consistent even when we accounted for competing risk of death (adjHR 0.78, 95% CI: 0.68–0.89, p < 0.001), as illustrated in Figure 5.

**FIGURE 6:**
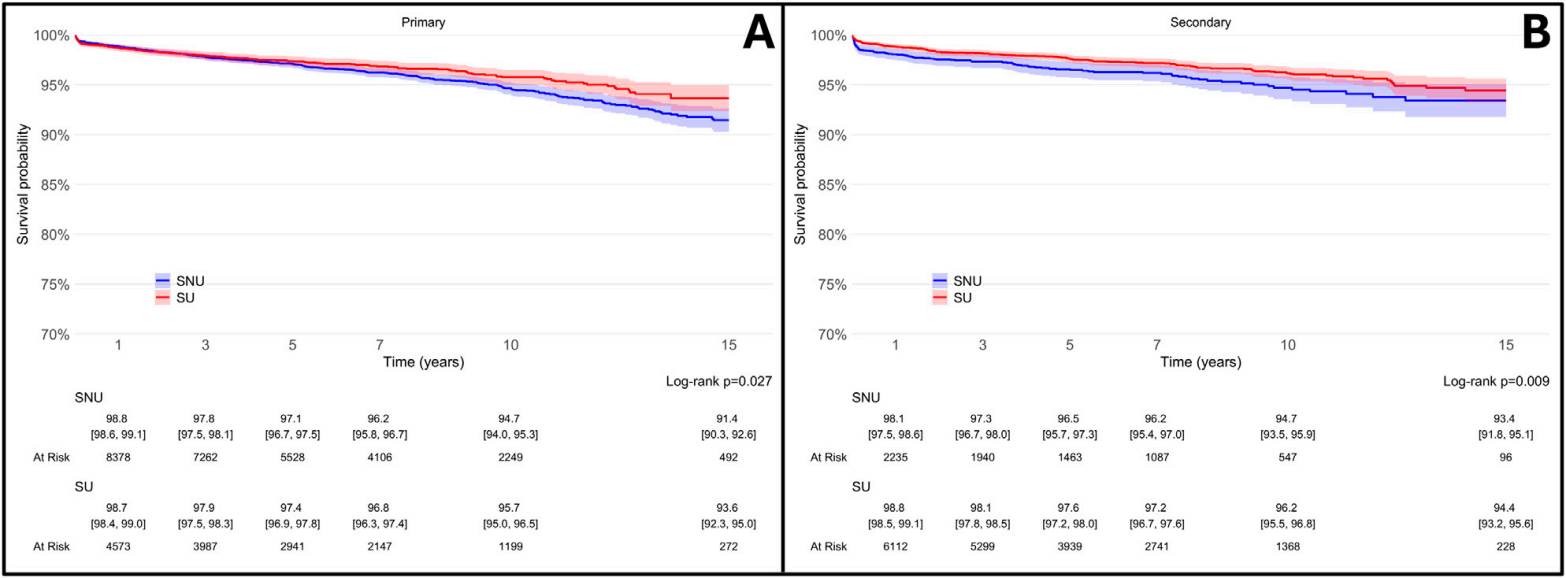
Survival rates of total hip arthroplasty (THA) in statin users (SU) vs. statin non-users (SNU) plotted according to the Kaplan-Meier method (main outcome: surgical revision) and stratified according to reason for statin prescription [primary cardiovascular prevention, **(A)**; secondary cardiovascular prevention, **(B)**].

Considering that the specific indication for statin therapy varies depending on whether it is prescribed for primary CVP (individuals with high cholesterol levels who have not yet experienced CHD, CeVD, PAD, or diabetes) or secondary CVP (patients with a known history of CHD, CeVD, PAD, or diabetes), and given its implications for statin potency and more aggressive lipid targets, we conducted a secondary analysis stratified by the indication for statin use (i.e., primary or secondary CVP). In both groups, SU consistently exhibited a lower likelihood of undergoing THA revision, as shown in Figure 6.

While the lipid-lowering efficacy of statins is determined by their relative potency and dosage, some cholesterol-independent pharmacodynamic effects are influenced by lipophilicity that implies differential cellular membrane permeability (Kang et al., 2022).

**FIGURE 7:**
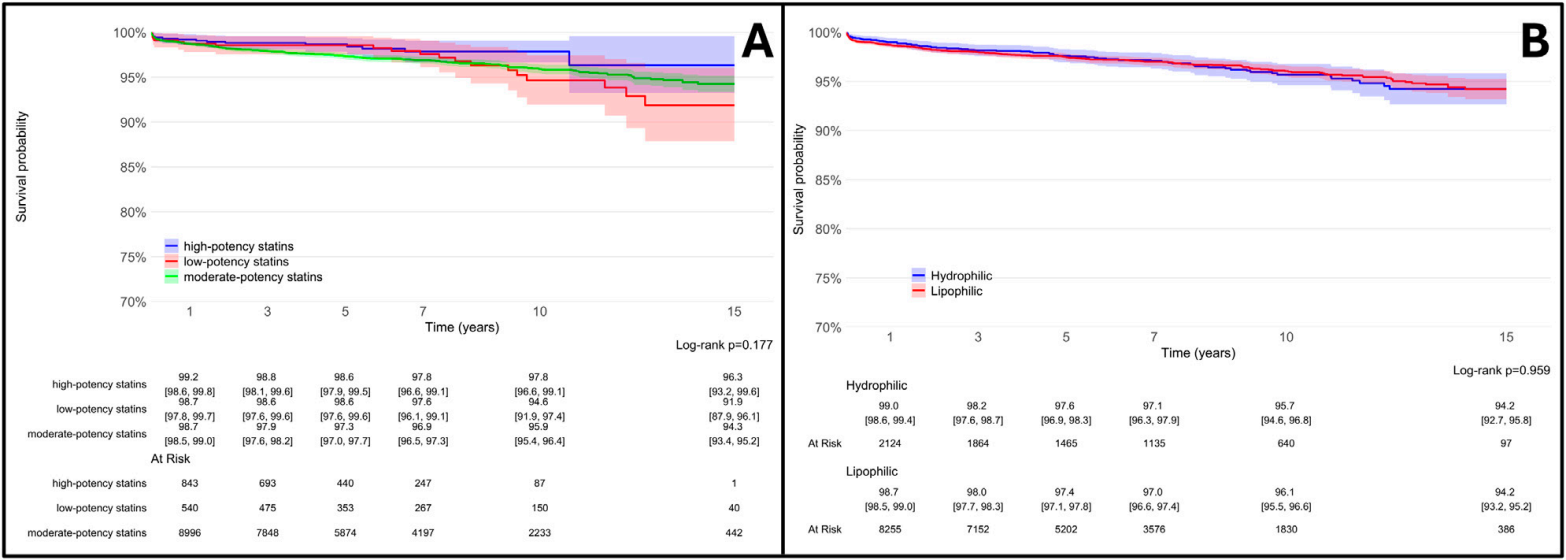
Survival rates of total hip arthroplasty (THA) in statin users (SU) vs. statin non-users (SNU) plotted according to the Kaplan-Meier method (main outcome: surgical revision) and stratified according to reason to statin potency [high, moderate or low potency - **(A)**] and lipophilicity [lipophilic or hydrophilic, **(B)**].

To account for these properties, we conducted additional secondary analyses, which revealed no significant differences in THA survival among patients stratified by statins potency or lipophilicity (Figure 7).

## 4 Discussion

In this retrospective observational study, we found that patients with hip OA who underwent THA and were receiving statins before surgery (SU group) had a 24% reduced risk of implant revision during a 15-year follow-up compared to a PS-matched cohort of individuals who were not receiving statins (SNU group). This effect appeared to be consistent, irrespective of the reason for statin prescription (namely, primary or secondary CVP) and was more evident in males.

Our study revealed a strong association between statin use and the survival of THA that remained consistent in different secondary analyses. Animal experiments have suggested that statins may enhance the integration of prostheses after arthroplasty (von Knoch et al., 2005b), a finding supported by a small case-control study where researchers observed significantly fewer cases of radiologically detectable femoral osteolysis in statin “ever-users” compared to “never-users” at 5 years after surgery timeframe (Lübbeke et al., 2013). Thillemann et al. conducted a registry-based study in Denmark, which indicated that individuals treated with statins and undergoing THA had a reduced risk of arthroplasty revision (Thillemann et al., 2010). This observation was further supported by Lalmohamed et al. that analyzed data from both the Danish and UK populations, although the effect was somewhat smaller (Lalmohamed et al., 2016). Our study not only confirms these findings in a large Italian cohort, but also adds valuable insights compared to previous studies.

First, our registry collects clinical data from patients before THA, eliminating the need to rely solely on administrative records. This allowed us to evaluate additional variables, i.e., BMI, and use this information to account for an important confounding factor in THA outcomes other than statin prescription, namely, obesity (Carender et al., 2023; Yumuk et al., 2015).

Another key advantage of our study is that we performed secondary analysis stratified according to the indication for statin treatment. We found that SU showed a reduced occurrence of THA revision compared to SNU across primary and secondary CVP. This finding is intriguing since primary and secondary CVP patients treated with statins represent two distinct populations in terms of physical performance, lifestyle habits, and comorbidities (Johnsen et al., 2006; Jones et al., 2004). Indeed, consistently with the available literature, patients in the SU group had a significantly higher prevalence of obesity, diabetes, CHD, CeVD and PAD as isolated or aggregated comorbidity. Despite it is known that subjects suffering from diabetes (King et al., 2013) or multiple comorbidities (Lakomkin et al., 2017) have an increased risk of THA revision, in our study SU patients had improved long-term THA survival. In our opinion, this finding further strengthen our results and suggest that the benefit of statins could be underestimated, given that multiple comorbidity could negatively affect THA survival, exerting a mitigating effect on the observed effect size (Sayed-Noor et al., 2019).

Furthermore, the intensity of lipid-lowering therapy is generally higher in secondary CVP patients (Mach et al., 2020), and many statins effects are known to be intensity-related, especially in cardiovascular prevention (Mach et al., 2020) but also for other hypothesized pleiotropic effects such as vascular protection during revascularization procedures (Athyros et al., 2015), cancer (Rodríguez-Miguel et al., 2022) and dementia prevention (Olmastroni et al., 2022). Our data demonstrated a consistent statins effect across primary and secondary CVP patients, with no significant differences between high, moderate or low-potency statins, suggesting a class effect of these drugs without a specific potency threshold for their effectiveness. This is particularly noteworthy, as it is in contrast with some evidence that has suggested dose-response relationships between statins and bone metabolism, particularly regarding cholesterol levels (Zheng et al., 2020). The class effect of statins is also supported by the evidence that the cohort of Thillemann and al. was treated for 77% with a low potency statin as simvastatin and showed a 66% reduced risk for SU to undergone THA revision during an average follow-up of 4.4 years (Thillemann et al., 2010). Interestingly, SU showed fewer THA revisions in males but not in females, suggesting a potential sex-specific effect of statins. This is a novel finding, as previous studies had not explored this hypothesis. It is well-established that bone metabolism exhibits sex (Khosla and Monroe, 2018) and gender (Violi et al., 2021) differences, making post-menopausal females more susceptible to certain bone diseases than males. Moreover, in terms of the effects of statin therapy on cardiovascular outcomes, sex (Petretta et al., 2010) and gender difference (Raparelli et al., 2017) have already been identified, with a greater clinical benefit of statin therapy observed in males. Therefore, the effects of statins on bone might share some sex-specific characteristics with their effects on cardiovascular outcomes. Lastly, despite *in vitro* studies suggesting that statin effects on bone may be peculiar to lipophilic statins (Oryan et al., 2015), our study did not provide support to this hypothesis as there was no difference based on statins lipophilicity.

In conclusion, statin treatment appears to have a protective effect on THA survival, particularly in males. Despite the seemingly distinct nature of bone metabolism and cardiovascular function, they share many pathways and risk factors for disease development (Vaiciuleviciute et al., 2021). For example, vascular endothelial function, inflammation, and extracellular matrix metabolism are key features in the development of both atherosclerosis (Gimbrone and García-Cardeña, 2016; Kong et al., 2022) and osteogenesis (Peng et al., 2020). Statins are known to modulate both mechanisms (Oesterle et al., 2017; Satny et al., 2021) as well as osteogenesis (Oryan et al., 2015) and may contribute to implants osteointegration (Von Knoch et al., 2005a). Therefore, it is conceivable that statin treatment creates a bone and systemic environment favorable for implant acceptance and long-term osteointegration, with a more pronounced effect in males.

However, this study has some limitations. First, as an observational study, it cannot establish a causal relationship between statin use and THA survival. The findings should therefore be interpreted as hypothesis-generating, encouraging further longitudinal studies specifically designed to assess the impact of statins on orthopedic surgery outcomes. Statin users were identified through administrative data, meaning a small subset of patients who purchased statins without SSN reimbursement may have been misclassified as SNU. However, this is unlikely in Italy, where long-term therapies are universally accessible under full SSN coverage. Additionally, the lack of data on blood cholesterol and lipoprotein levels makes it uncertain whether the observed effect is driven by statin therapy itself, lipid reduction, or the well-documented pleiotropic effects of statins on inflammation and bone metabolism. Similarly, comorbidities were assessed using registry data and hospital discharge records, with accuracy depending on the information recorded by healthcare providers in discharge forms. Diabetes status was determined through ICD codes and drug prescriptions—a method that, despite its challenges, likely captures most diabetes cases in Italy, where medical tests and treatments are routinely provided through the SSN. Moreover, all patients underwent comprehensive medical and anesthetic evaluations before THA surgery, which should have identified undiagnosed diabetes when present. Finally, despite adjustments for diabetes and other key comorbidities (obesity, CHD, CeVD, and PAD) and the use of PS matching, residual confounding remains due to unmeasured factors such as lifestyle, physical activity, bone health, and concurrent pharmacological treatments.

Regarding physical activity, it can be speculated that patients on statins may have a higher morbidity burden and poorer physical status, which could limit their activity levels. As a result, the prosthetic implant may not be fully utilized, potentially reducing wear, or the physical condition of these patients may hinder revision surgery after many years.

On the other hand, the strength of this study is the large sample size and long follow-up period, as well as its focus on a more recent cohort compared to previous studies. Importantly, the study only included and matched patients who were already taking statin before THA, eliminating potential confounding factors related to the timing of statin initiation post-surgery. Finally, various baseline data in our study were collected directly by the surgeon rather than extracted from databases.

## 5 Conclusion

In conclusion, our findings suggest that preoperative statin treatment may be associated with a lower rate of THA revision in patients with OA, particularly in males. This finding remains valid regardless of the rationale behind the prescription of lipid-lowering treatment and is independent from the main pharmacokinetic and pharmacodynamic properties of the statin received.

The current study provides further strength to the growing body of evidence supporting the hypothesis that these cost-effective drugs may have pleiotropic effects including bone anabolic properties. As a result, concomitant statin medication for cardiovascular prevention in the preoperative period may be regarded as a favorable prognostic factor when weighing the risk-benefit profile of patients undergoing THA.

## Level of evidence

Prognostic Level III.

## Data Availability

Publicly available datasets were analyzed in this study. This data can be found here: https://ripo.cineca.it/authzssl/Reports.html.

## Abbreviations

adjHR, age- and sex-adjusted hazard ratio; ATC, Anatomical Therapeutic Chemical classification system; BMI, Body mass index; CeVD, Cerebrovascular disease; CHD, Coronary heart disease; CVP, Cardiovascular prevention; HMG-CoA, 3-hydroxy-3-methylglutaryl coenzyme A; ICD-9, International Classification of Diseases; ISS, Istituto Superiore di Sanità; OA, Hip osteoarthritis; PAD, Peripheral artery disease; PS, Propensity score; PTA, Pharmaceutical Territorial Assistance; RIPO, Emilia-Romagna Orthopedic Arthroplasty Implants Register; SNU, Statin non-user; SSN, Italian National Health System; SU, Statin user; THA, Total hip arthroplasty.
